# Dissecting Quantitative Trait Loci for Spot Blotch Resistance in South Asia Using Two Wheat Recombinant Inbred Line Populations

**DOI:** 10.3389/fpls.2021.641324

**Published:** 2021-03-04

**Authors:** Chandan Roy, Navin C. Gahtyari, Xinyao He, Vinod K. Mishra, Ramesh Chand, Arun K. Joshi, Pawan K. Singh

**Affiliations:** ^1^Department of Plant Breeding and Genetics, Bihar Agricultural University, Sabour, India; ^2^ICAR–Vivekanand Parvatiya Krishi Anushandhan Sansthan, Almora, India; ^3^International Maize and Wheat Improvement Center (CIMMYT), Texcoco, Mexico; ^4^Department of Genetics and Plant Breeding, Institute of Agricultural Sciences, Banaras Hindu University, Varanasi, India; ^5^Department of Mycology and Plant Pathology, Institute of Agricultural Sciences, Banaras Hindu University, Varanasi, India; ^6^CIMMYT-India/Borlaug Institute for South Asia, New Delhi, India

**Keywords:** *Bipolaris sorokiniana*, SNPs, bi-parental mapping, DArTSeq, wheat QTLs for SB resistance

## Abstract

Spot blotch (SB) disease causes significant yield loss in wheat production in the warm and humid regions of the eastern Gangetic plains (EGP) of South Asia (SA). Most of the cultivated varieties in the eastern part of SA are affected by SB under favorable climatic conditions. To understand the nature of SB resistance and map the underlying resistant loci effective in SA, two bi-parental mapping populations were evaluated for 3 years, i.e., 2013–2015 for the BARTAI × CIANO T79 population (denoted as BC) and 2014–2016 for the CASCABEL × CIANO T79 population (CC), at Varanasi, Uttar Pradesh, India. DArTSeq genotyping-by-sequencing (GBS) platform was used for genotyping of the populations. Distribution of disease reaction of genotypes in both populations was continuous, revealing the quantitative nature of resistance. Significant “genotype,” “year,” and “genotype × year” interactions for SB were observed. Linkage map with the genome coverage of 8,598.3 and 9,024.7 cM in the BC and CC population, respectively, was observed. Two quantitative trait loci (QTLs) were detected on chromosomes 1A and 4D in the BC population with an average contribution of 4.01 and 12.23% of the total phenotypic variation (PV), respectively. Seven stable QTLs were detected on chromosomes 1B, 5A, 5B, 6A, 7A, and 7B in the CC population explaining 2.89–10.32% of PV and collectively 39.91% of the total PV. The QTL detected at the distal end of 5A chromosome contributed 10.32% of the total PV. The QTLs on 6A and 7B in CC could be new, and the one on 5B may represent the *Sb2* gene. These QTLs could be used in SB resistance cultivar development for SA.

## Introduction

Spot blotch (SB) disease caused by *Cochliobolus sativus* (Ito and Kuribayashi) Drechsler ex Dastur [anamorph *Bipolaris sorokiniana* (Sacc.) Shoemaker] is considered a significant disease of wheat (*Triticum aestivum* L.) in South Asia (SA) (Gupta et al., 2018). High temperature and humidity favor the disease development in the warmer wheat growing areas of the eastern Gangetic plains (EGP), particularly in Bangladesh (Siddique et al., 2006), Nepal (Sharma et al., 2007b), and eastern India (Joshi et al., 2007a). The long-established practice of rice–wheat cropping system in the EGP delays the sowing of wheat crop that provides a congenial humid and warm environment for the SB development in the later stages of crop growth. Average yield loss due to SB ranges from 15 to 20%, but under favorable environment, up to 87% yield loss has been observed in susceptible genotypes (Hetzler et al., 1991). Delayed seeding of wheat in the EGP resulted on an average loss of 30% yield due to complex foliar blights, especially SB (Duveiller et al., 2005). Trait association analysis revealed that days to heading (DH) and plant height (PH) often showed a negative correlation with SB severity (Singh et al., 2015). Several attempts have been made including cultural practices and chemical application to control SB, but none of them was completely successful. Integrated disease management using host resistance, chemical control, and cultural practices is considered most effective in managing the disease (Joshi and Chand, 2002).

Except for a recent attempt (Kumar et al., 2019), no host immunity has been observed for SB, and the best released cultivars are only partially resistant. SB resistance is under polygenic control, with quantitative trait loci (QTL) of various phenotypic effects; hence, the progress of cultivar development is relatively slow. Genetic studies for SB resistance have identified multiple QTLs, of which four with major effects have been nominated, i.e., *Sb1* through *Sb4.* The *Sb1* is located on chromosome 7DS flanked by the markers *Xgwm1220* and *Xgwm295*, being co-located with the leaf rust resistance locus *Lr34* having pleiotropic effects on resistance to yellow rust (*Yr57*), powdery mildew (*Pm8*), and leaf tip necrosis (Ltn+) (Lillemo et al., 2013). The *Sb2* (*Qsb.bhu-5B*) has been mapped on chromosome 5BL flanked by the simple sequence repeat (SSR) markers *Xgwm639* and *Xgwm1043* (Kumar et al., 2015). The third gene, *Sb3*, was mapped on chromosome 3BS (Lu et al., 2016), being in the same region where two previously reported QTLs *Qsb.bhu 3B* and *Qsb.cim 3B* reside. Recently, the *Sb4* gene has been mapped on the long arm of chromosome 4B, where 21 putative genes were predicted (Zhang et al., 2020). QTLs with minor effects are also important for SB resistance since stacking such QTLs significantly reduced SB severity (Singh et al., 2018). Multiple minor QTLs have been mapped on 1A, 1B, 1D, 2B, 2D, 3A, 3B, 4A, 5A, 5B, 6A, and 7A (Gurung et al., 2014; Zhu et al., 2014; Singh et al., 2016; Bainsla et al., 2020).

Germplasm development for SB resistance started in the 1980s, which led to the identification of several wheat genotypes with variable resistance like Saar, Yangmai 6, Shanghai 4, M3, Chirya 1, Chirya 3, Chirya 7, and SYN1 (Ibeagha et al., 2005). Looking at the growing incidence of SB in SA, CIMMYT developed a special nursery in 2009 for SA named CSISA-SB, under the Cereal System Initiative for South Asia (CSISA) project. The purpose was to share CIMMYT breeding lines with SB resistance and good agronomic performance with the researchers of other countries and to test the nursery over various locations. This nursery was renamed Helminthosporium Leaf Blight Screening Nursery (HLBSN) in 2015 and distributed beyond SA to South American and African countries like Brazil, Bolivia, Paraguay, and Zambia, where SB is of major concern. The SB screening platform of CIMMYT in Mexico is located at Agua Fria, where the climate is similar to SA, providing strong support in the selection of SB-resistant genotypes for SA (Singh et al., 2015).

In SA, the Varanasi center of India has been identified as one of the most suitable sites for the evaluation of SB; it has a close similarity with the climatic conditions of Bhairahawa and Rampur of Nepal (Joshi et al., 2007a). Previously, four bi-parental mapping populations were evaluated at Agua Fria, Mexico, and their underlying QTLs have been identified (Singh et al., 2018; He et al., 2020). In the present study, we evaluated two of those four mapping populations at Varanasi, India, to determine the resistant QTLs effective under the SA environment.

## Materials and Methods

### Plant Materials

Two SB-resistant lines BARTAI (BABAX/LR 42//BABAX/3/ERA F 2000) and CASCABEL (SOKOLL//W15.92/WEEBILL1) identified in the previous experiments were crossed with a common susceptible parent CIANO T79 (BUCKY/(SIB)MAYA-74/4/BLUEBIRD//HD-832.5.5/OLESEN/3/CIANO-67/PENJAMO-62) to develop two bi-parental mapping populations (Singh et al., 2018; He et al., 2020). Recombinant inbred lines (RILs) were generated following the single seed descent method from F_2_ generation of the cross BARTAI × CIANO T79 (BC population) and CASCABEL × CIANO T79 (CC population) at CIMMYT, Mexico. Field experiments were conducted using a total of 231 RILs of BC and 226 RILs of CC in F_2:7_ generation along with the parents constituting the populations, and genotypes Chirya 3 and Sonalika were included as resistant and susceptible check, respectively.

### Field Experiments

Field evaluation was carried out at the experimental station of Banaras Hindu University (BHU, 25.2°N, 83.0°E), Varanasi, India, in the years 2012–2013 (denoted as 2013), 2013–2014 (2014), and 2014–2015 (2015) for the BC population, and in the years 2013–2014 (2014), 2014–2015 (2015), and 2015–2016 (2016) for the CC population. Sowing was done in December, under late sown conditions to expose the crop to high temperature and humidity at the later stage of crop growth, which favors SB disease development. The experiments were conducted in a randomized complete block design with two replications, where each entry was sown in 2-m double rows spaced 25 cm apart, with a plant-to-plant distance of 5 cm.

### Inoculation Method and SB Assessment

The pure culture of *B. sorokiniana* (isolate HD 3069/MCC 1572) was maintained using potato dextrose agar (PDA) medium (Chand et al., 2003). The pathogen was mass multiplied on previously soaked and autoclaved sorghum grains, which was kept under room temperature for at least 6 weeks. Spore suspension culture was prepared at a concentration of 1 × 10^4^ spores ml^–1^. To create an artificial epiphytotic condition, the spore suspension was inoculated at the heading stage [Zadok’s growth stage (GS) 55] in the evening time. Light irrigation was given after inoculation to maintain high humidity for disease development.

Disease scoring was done for three subsequent growth stages at the beginning of anthesis (GS 63), after completion of anthesis (GS 69), and late milking (GS 77) using a double-digit (00–99) scale as prescribed by Saari and Prescott (1975). The first digit (D1) measured disease progress in PH and the second digit (D2) measured the disease severity in terms of the proportion of infected leaf area. The percentage of disease severity for each score was measured as:

Severity(%)=(D1/9)×(D2/9)×100

Area under disease progress curve (AUDPC) was calculated using percent of severity estimations corresponding to disease rating as:

$$\mathrm{AUDPC}=\sum_{1}^{n}{[\{(Y}_{i}+Y_{i+1})/2\}\times \{t_{\left(i+1\right)}-t_{i}\}]$$

where

*Y*_*i*_ = disease level at the time *t*_*i*_.

*Y*_(_*_*i*_*_+__1)_ = disease level at time *t*_*i*__+__1_.

*t*_*i*__+__1_ – *t*_*i*_ = time difference in days between two disease scores.

*n* = number of readings.

Area under disease progress curve average of two replications in a single year and mean AUDPC across all 3 years were used for QTL analysis. DH and PH were also measured to determine their association with SB.

### Statistical Analysis

Analysis of variance (ANOVA) and Pearson correlation coefficients were calculated using statistical software OPSTAT^1^. Marker-based narrow sense heritability was calculated with the “heritability” package of R (Kruijer et al., 2015).

### Genotyping and Linkage Analysis

Genomic DNA was isolated using the cetyltrimethylammonium bromide (CTAB) method from each entry including the parental lines of respective populations. Genotyping was carried out using the DArTSeq genotyping-by-sequencing (GBS) platform (Li et al., 2015) at the Genetic Analysis Service for Agriculture (SAGA) in Guadalajara, Mexico. Several gene-based markers and D-genome-specific single-nucleotide polymorphism (SNP) markers using “Kompetitive allele-specific PCR” (KASP) were also used. QTL analysis was carried out using an integrated software package ICIMapping version 4.1 (Meng et al., 2015). Monomorphic markers, markers with missing value >20%, and minor allele frequency <30% were removed from QTL analysis. Chromosome anchoring was done for each marker as per the GBS map described by Li et al. (2015). Linkage groups (LGs) were constructed using the MAP function in the ICIMapping software version 4.1, with the LOD threshold set at 15 and the rest parameters at default.

Quantitative trait loci mapping was performed using the BIP function of ICIMapping, where interval mapping was first carried out to identify significant QTLs and after that inclusive composite interval mapping was performed to identify more robust QTLs. QTL mapping was also carried out after adjusting for DH and PH. Adjusted mean was calculated by the software Multi-Environment Trial Analysis with R (META-R) version 6.0 using DH and PH as cofactors. A QTL was considered significant when it exceeded the LOD threshold of 3.4 (1,000 permutations at α = 0.05) for BC and LOD of 3.6 for CC populations in at least one environment. However, QTL with an LOD value of 2.5 or above appearing in more than one environment was also considered as significant. To draw the LGs and LOD curve, software MapChart v. 2.3 (Voorrips, 2002) was used.

## Results

### Phenotyping for SB Resistance

**FIGURE 1:**
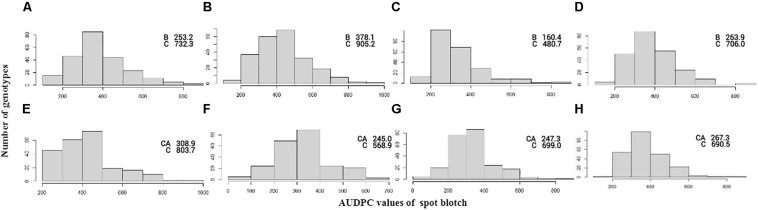
Frequency distribution of AUDPC scores in the BC population in the year 2013 **(A)**, 2014 **(B)**, 2015 **(C)**, and mean **(D)** and CC population in the year 2014 **(E)**, 2015 **(F)**, 2016 **(G)**, and mean **(H)**. AUDPC score of parents BARTAI, CASCABEL, and CIANO T79 is denoted as B, CA, and C.

Significant genetic variation was observed for SB among the genotypes in both the BC and CC populations. Effects of climatic fluctuations across years on SB development were revealed by significant variation in “year” and “genotype × year” interaction effects; however, for the CC population, the latter effect was non-significant (Table 1). Disease pressure was maximum in the year 2014 and least in 2015 for both populations. A similar trend was also observed for Sonalika and Chirya 3, the susceptible and resistant checks, respectively. Continuous distribution of genotypes for SB resistance in different years and their mean were observed (Figure 1). Transgressive segregants for resistance and susceptibility were obtained in both the populations. Twenty-three resistant transgressive segregants were found in the BC population, out of which seven genotypes performed better than the resistant check Chirya 3, whereas in the CC population, 55 genotypes showed higher resistant than CASCABEL, out of which 10 genotypes were better than Chirya 3.

**TABLE 1:** Analysis of variance and heritability estimates of spot blotch resistance in BARTAI × CIANOT79 (BC) and CASCABEL × CIANOT79 (CC) populations.

Source of variation	DF	Mean squares	*F* calculated	Significance	Heritability
**BC population**					
Year	2	13,488.82	5,748.176	<0.0001	
Rep (year)	3	2.34	2.46	NS	
Genotype	230	58.91	2.784	<0.0001	0.61
Year × genotype	460	21.15	21.162	<0.0001	
Pooled error	690	0.95			
**CC Population**					
Year	2	103.37	58.033	<0.01	
Rep (year)	3	1.78	1.78	NS	
Genotype	225	28.58	126.553	0.0001	0.73
Year × genotype	450	0.22	0.226	NS	
Pooled error	675	1.00			

*NS, non-significant.*

Moderate heritability estimates for SB in BC (0.61) and CC (0.73) were recorded (Table 1). DH and PH were mostly negatively correlated with SB. PH was found to be more closely associated with SB than DH as exhibited in the significantly negative association across all the environments in both populations (Table 2).

**TABLE 2:** Pearson correlation coefficient analysis of spot blotch resistance with days to heading and plant height in BARTAI × CIANOT79 (BC) and CASCABEL × CIANOT79 (CC) populations.

Year	2013	2014	2015
**BC population**			
Days to heading	−0.430**	0.033^NS^	−0.111^NS^
Plant height	−0.432**	−0.344**	−0.230**
**CC population**			
Year	2014	2015	2016
Days to heading	−0.096^NS^	−0.194**	−0.121^NS^
Plant height	−0.296**	−0.299**	−0.334**

**P < 0.001; ^NS^non-significant.

### Genotyping and Linkage Analysis

Out of 18,000 GBS markers scored in both populations, 3,174 and 3,197 high-quality non-redundant markers in BC and CC populations, respectively, were screened out for linkage analysis and QTL mapping (Supplementary Tables 1, 2). Both populations contained 21 large LGs representing all the 21 wheat chromosomes, as well as a few fragmented LGs that were not used in subsequent analysis. The linkage map of the BC population covered 8,598.3 cM with an average distance of 2.71 cM between markers, while in the CC population, 9024.21 cM was covered with an average distance of 2.82 cM between markers. All chromosomes were in good coverage with the least length of 199.22 cM for 1D in the BC population and 223.69 cM for 4D in the CC population. The coverage for chromosomes of A and B sub-genome was better than that of D genome in both populations (Supplementary Tables 1, 2).

### QTL Identification for SB Resistance

**FIGURE 2:**
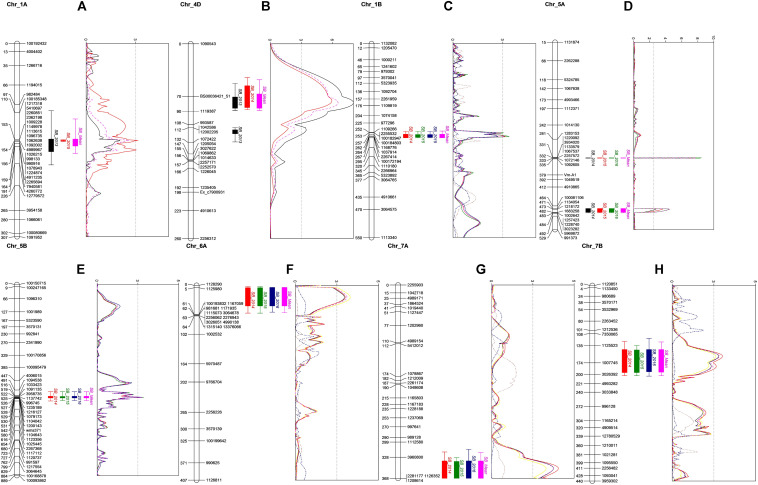
QTL profiles for SB in BC population on chromosomes 1A **(A)** and 4D **(B)**, and in CC population on chromosomes 1B **(C)**, 5A **(D)**, 5B **(E)**, 6A **(F)**, 7A **(G)**, and 7B **(H)**. Genetic distance in cM is presented in the left side of each chromosome. A LOD threshold of 2.5 is indicated by the vertical dashed line.

The QTL with the largest phenotypic variation explained (PVE) in the BC population was detected on chromosome 4D, with a mean PVE of 12.23% (Table 3). This QTL was found to be associated with PH. Another QTL was detected on chromosome 1A with the mean phenotypic effect of 4%. Two additional QTLs with minor effects were detected on 4D in 2013 and 5B in 2015 (Table 3 and Figure 2). All the resistance alleles of the QTLs in BC population were contributed by the resistant parent BARTAI. When PH was used as a covariate, the effect of the QTL reduced; in addition, few other QTLs on 4B, 5B, and 6B chromosomes were detected (Supplementary Table 3).

**TABLE 3:** Quantitative trait loci (QTLs) identified for SB in the BARTAI × CIANO T79 (BC) and CASCABEL × CIANO T79 (CC) populations and their associated QTLs in literature.

Chromosome	Position (cM)	Left marker^b^	Right marker	LOD^c^	2013^d^	2014	2015	Mean	Source of resistance	Associated QTLs in literature
**BARTAI × CIANO T 79 (BC)**										
1A	153.79–155.61	4989967	1026215	2.86	2.31	–	3.97	**4.01**	BARTAI	Zhu et al., 2014
4D^a^	70.49–90.31	BS00036421_51	1119387	6.40	**10.45**	**9.53**	**–**	**12.23**	BARTAI	Singh et al., 2018; He et al., 2020
4D	112.48–131.93	12002205	1072422	3.46	4.49	–	–	–	BARTAI	
5B	135.84–138.59	9724385	2267710	2.59	–	–	**4.75**	–	BARTAI	Jamil et al., 2018; He et al., 2020
Percentage of accumulated phenotypic variation								16.24		
**CASCABEL × CIANO T 79 (CC)**										
1B	261.53–263.82	1168776	1037914	3.11	3.11	2.90	2.79	2.89	CIANO T79	Singh et al., 2018; Bainsla et al., 2020; He et al., 2020
5A^a^	331.49–332.06	1067537	2257572	7.72	**12.62**	**8.93**	**11.26**	**10.32**	CASCABEL	Ayana et al., 2018; Singh et al., 2018; Bainsla et al., 2020; He et al., 2020
5A	472.56–481.59	1218172	1683258	4.51	**4.59**	**4.13**	**5.71**	**5.89**	CIANO T79	
5B	522.08–525.15	3958735	1137742	2.74	**5.32**	**5.77**	**4.02**	**5.56**	CASCABEL	Kumar et al., 2009, 2010, 2015; Jamil et al., 2018; He et al., 2020
6A	5.08–61.14	1125980	100193832	3.10	5.79	5.24	**6.48**	**5.70**	CASCABEL	
7A	328.34–367.52	1126352	1208614	3.74	**5.45**	**5.32**	**5.19**	**5.27**	CIANO T79	
7B	134.52–173.55	1125523	1007745	2.82	4.56	4.18	4.31	4.28	CIANO T79	Singh et al., 2016; Ayana et al., 2018; Singh et al., 2018
Percentage of accumulated phenotypic variation								39.91		

*^a^Number used to distinguish QTLs exceeded the LOD threshold of 3.4 for BC and 3.6 for CC population.^b^Sequence information of the markers is available in Supplementary Table 4.^c^LOD values of QTLs in the mean population were used.^d^Percentage of phenotypic variation explained (PVE) is provided; QTLs in bold remained significant after the ICIM algorithm.*

**FIGURE 3:**
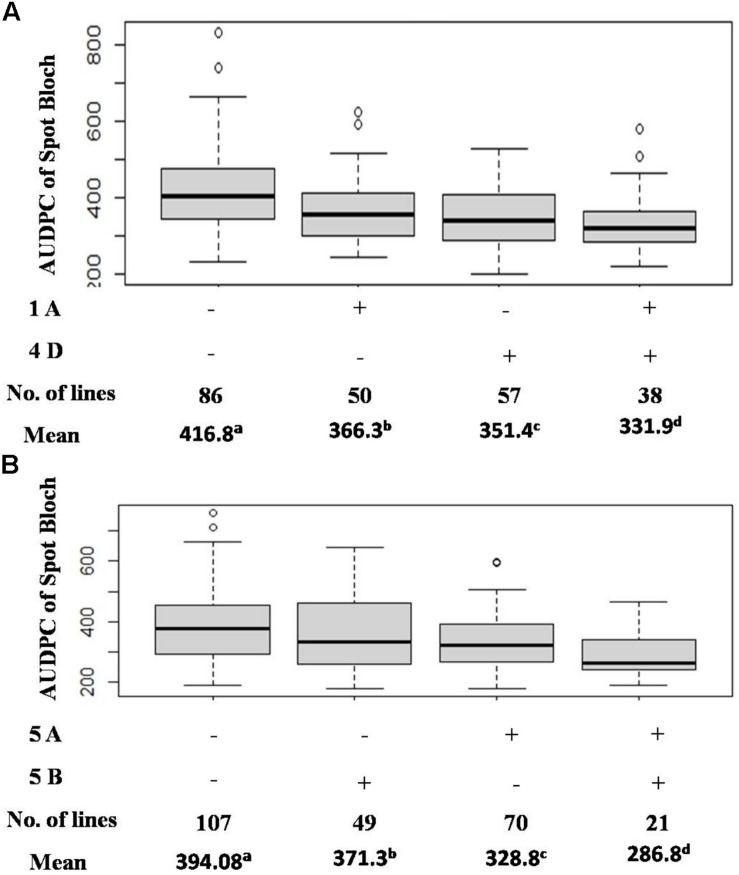
Effects of QTL combinations in reducing AUDPC in the BC **(A)** and CC **(B)** population.

Seven QTLs were detected in the CC population, altogether explaining 39.91% of phenotypic variation (PV). The QTLs were detected on six chromosomes (1B, 5A, 5B, 6A, 7A, and 7B) with the mean PVE ranging from 2.89 to 10.32%. The resistance alleles of QTLs on 1B, 5A (proximal), 7A, and 7B were contributed by CIANO T79, whereas those of QTLs on 5A (distal), 5B, and 6A were contributed by CASCABEL. Out of 55 resistant transgressive segregants, 40 were carrying at least one QTL contributed by CIANO T79. The major QTL was detected on the distal end of chromosome 5A with a PV ranging from 8.93 to 12.62% in 2015 and 2014, respectively, with a mean PV of 10.32%. The effect of QTLs in the reduction of AUDPC appeared to be additive in nature (Figure 3).

## Discussion

Due to global warming and climate change, wheat production is predicted to be adversely affected (Rosyara et al., 2010). Higher temperature combined with rains during the grain filling stages increases the chances of SB in EGP, resulting in significant yield losses. Since the vulnerability of the wheat crop to SB increases when temperature exceeds 26°C at post-anthesis stage (Chaurasia et al., 2000), sowing in this study was delayed, exposing the populations under higher disease pressure. Joishi et al. (2002) reported that the resistance is independent of plant growth stage as there is appearance of substantial proportion of resistance in tall and dwarf progenies obtained from resistant tall × susceptible dwarf and in early and late progenies from resistant tall × susceptible early cross, respectively. However, to avoid the influence of growth stages on the disease appearance, scoring was done at different growth stages. The present observation on the negative correlation of DH and PH with SB has shown similarity with the previous reports from Agua Fria (Singh et al., 2018) and SA (Singh et al., 2016). Negative association of DH and PH with SB implies the selection of late and tall genotypes for better SB resistance, but such cultivars are not suitable for high rainfall and warmer regions like EGP of SA (Joshi et al., 2007b). Fortunately, this association has been broken, and several early maturing SB resistance cultivars have been identified for warm climatic conditions of SA (Sharma et al., 2004; Joshi et al., 2007b).

All experiments in the current study exhibited a typical quantitative inheritance of SB resistance with strong genotype × environment (G × E) interactions, which has also been reported in earlier studies constituting Indian germplasm, CIMMYT derivatives, and Afghan landraces (Kumar et al., 2016; Singh et al., 2018; Bainsla et al., 2020). Strong G × E interaction has always been a concern for plant breeders as it influences varietal adaptation across the environments. QTL × environment interaction, a component of G × E interaction, affects the efficiency of marker-assisted selection. Identification of QTLs across the location and year helps breeders in design and implementation of breeding strategies for the improvement of complex traits for adaptation in specific or mega environment. Veldboom and Lee (1996) reported that QTLs identified in the mean and across the environment are of major importance. In our study, two stable QTLs on chromosomes 1A and 4D were detected in the BC population, with the latter explaining major PVs. In a previous report, when the same population was evaluated at CIMMYT’s Agua Fria station, a QTL on 4D chromosome was mapped at the same chromosomal region but was significant only in 1 year, having a PVE of 3.6% (Singh et al., 2018), implying a stronger influence of PH on SB resistance in SA. The QTL on 1A was not found in Singh et al. (2018) and thus might be specific only to SA environments. In a previous study, Zhu et al. (2014) identified a QTL on 1A, being close to the QTL identified in the present study.

A QTL on chromosome 5A delimited by the flanking markers *1067537–2257572* was significant in all 3 years explaining major PV in CC population. The same QTL was identified in the previous studies at Agua Fria but with higher phenotypic effects that reduced significantly when adjusted for PH and DH (Singh et al., 2018; He et al., 2020). In the current study, the QTL was identified 47.0 cM distance away from the vernalization locus *Vrn-A1*; also, the effect of QTL remained significant in all the 3 years when DH and PH were used as covariates. The allele *vrn-A1* responsible for late maturity was associated with SB resistance, escaping the disease due to delayed phenology. However, the possibility of the presence of a SB resistance QTL in close proximity of *Vrn-A1* cannot be excluded (Singh et al., 2018; He et al., 2020) due to remnant effects of the QTL after adjusting for DH. Similarly, Bainsla et al. (2020) reported a marker 0.8 Mb away from *Vrn-A1* that is responsible for SB resistance. Likewise, Zhu et al. (2014) mapped a SB resistance QTL *QSb.cim-5A* at 30.3 cM away from *Vrn-A1*. The strong marker trait association would be useful for the selection of SB resistance QTL based on *Vrn-A1*.

The QTL identified on 5B chromosome in the CC population was also reported earlier when evaluated at Agua Fria (He et al., 2020). However, in the present study, the contribution of this QTL was lower than the previous report, ranging from 4.02 to 5.77% in different years of evaluation. Comparing the QTL position using the sequences of flanking markers through BLAST to the IWGSC RefSeq v1.0 genome sequence of Chinese Spring (CS), the position of this QTL coincides with the previously identified *Sb2* gene (He et al., 2020). The presence of *Sb2* was reported in resistant genotypes Yangmai 6 (Kumar et al., 2009), Ning8201 (Kumar et al., 2010) and CASCABEL (He et al., 2020). Additionally, this gene has been detected in an Afghan population (Bainsla et al., 2020), CIMMYT germplasm (Jamil et al., 2018), and a diverse germplasm panel with global origin (Gurung et al., 2014), suggesting that *Sb2* has been selected by breeders of different continents due to its positive effects on SB resistance. Recently, *Tsn1* on 5BL was identified as a sensitivity gene for the pathogen carrying the corresponding virulent gene *ToxA* (Friesen et al., 2018). It was suggested that *Tsn1* gene is the susceptibility gene of *Sb2* (Friesen et al., 2018), but He et al. (2020) proposed that the two genes might be different. A recent study of Indian *B. sorokiniana* population indicated that about 70% of the isolates carried *ToxA* (Navathe et al., 2019). The presence of *ToxA* in Mexican *B. sorokiniana* isolates (Wu et al., 2021) indicates the similarity in virulence factors of pathogenic population in Mexico and SA that reflects why the selections in Agua Fria are effective for SA.

Three QTLs on 6A, 7A, and 7B in the CC population were identified only in SA. Anchoring the flanking markers of the 6A and 7B QTLs in the CS reference genome indicated that they both reside at the distal end of their respective chromosomes. Earlier, SB resistance QTL was mapped on 6A (Sharma et al., 2007a) and 7B (Singh et al., 2016; Ayana et al., 2018) but at different positions, implying that those mapped in the current study might be new. However, the QTL on 7A might not be new, since QTLs with similar confidence intervals were reported in the KATH × CIANO T79 population (Singh et al., 2018), in a CIMMYT wheat panel (Jamil et al., 2018), as well as in the Afghan landrace collection (Bainsla et al., 2020).

The pathotype diversity of *B. sorokiniana* and some climatic difference among SA and Mexico environments possibly play a significant role in the identification of effective QTLs. In our study, QTLs specific to SA were detected in both populations, providing an opportunity for breeding SB resistance cultivars in SA. In CC, an average of 27.2% reduction in SB was observed when the 5A and 5B QTLs were combined, compared to a 42.3% reduction of SB at Agua Fria (He et al., 2020). Detection of QTLs on 5A and *Sb2* at both SA and Agua Fria indicated the potentiality of these genes in resistance breeding for SA, but their lower phenotypic effects for SB in SA environment indicates the role of other QTLs, like those on 4D, 6A, and 7A. Furthermore, validation of these QTLs and markers over multiple locations and years will provide not only more insight into the SB resistance in SA but also more robust markers for the development of SB-resistant cultivars targeting SA.

## Data Availability Statement

The original contributions presented in the study are publicly available. This data can be found here: https://hdl.handle.net/11529/10548547.

## Author Contributions

PS, AJ, and XH designed the research and provided the plant material. VM and RC conducted the field experiments. CR, NG, and XH analyzed the data. CR drafted the first version of the manuscript. All authors contributed to the writing of the manuscript.

## Conflict of Interest

The authors declare that the research was conducted in the absence of any commercial or financial relationships that could be construed as a potential conflict of interest.

## Footnotes

1 http://14.139.232.166/opstat/
