# Supplementary material for: Dissecting Quantitative Trait Loci for Spot Blotch Resistance in South Asia Using Two Wheat Recombinant Inbred Line Populations
Source: Front Plant Sci. 2021 Mar 4;12:641324. doi: 10.3389/fpls.2021.641324 (PMC7969869; doi:10.3389/fpls.2021.641324)
Supplement: Supplementary file 1 [file Data_Sheet_1.docx]

**Supplementary table S1**. Marker and linkage map statistics for the BARTAI x CIANO T79 population

| Chromosome | Number of markers | Genetic length (cM) | Average distance (cM) between markers |
| --- | --- | --- | --- |
| 1A | 157 | 307.07 | 1.96 |
| 1B | 252 | 558.67 | 2.22 |
| 1D | 51 | 199.22 | 3.91 |
| 2A | 206 | 690.27 | 3.35 |
| 2B | 243 | 328.15 | 1.35 |
| 2D | 41 | 515.54 | 12.57 |
| 3A | 147 | 600.84 | 4.09 |
| 3B | 216 | 587.23 | 2.72 |
| 3D | 21 | 354.46 | 16.88 |
| 4A | 142 | 359.47 | 2.53 |
| 4B | 103 | 429.48 | 4.17 |
| 4D | 18 | 260.06 | 14.45 |
| 5A | 203 | 442.28 | 2.18 |
| 5B | 291 | 369.61 | 1.27 |
| 5D | 37 | 219.60 | 5.94 |
| 6A | 163 | 254.45 | 1.56 |
| 6B | 282 | 393.55 | 1.40 |
| 6D | 46 | 397.29 | 8.64 |
| 7A | 213 | 352.46 | 1.65 |
| 7B | 256 | 428.50 | 1.67 |
| 7D | 86 | 550.10 | 6.40 |
| Whole Genome | 3174 | 8598.3 | 2.71 |

**Supplementary table S2**. Marker and linkage map statistics for the CASCABEL x CIANO T79 population

| Chromosome | Number of markers | Genetic length (cM) | Average distance (cM) between markers |
| --- | --- | --- | --- |
| 1A | 163 | 408.72 | 2.51 |
| 1B | 199 | 550.14 | 2.76 |
| 1D | 33 | 271.25 | 8.22 |
| 2A | 193 | 591.40 | 3.06 |
| 2B | 385 | 670.00 | 1.74 |
| 2D | 43 | 278.76 | 6.48 |
| 3A | 214 | 544.9 | 2.55 |
| 3B | 192 | 424.65 | 2.21 |
| 3D | 105 | 274.26 | 2.61 |
| 4A | 163 | 352.48 | 2.16 |
| 4B | 20 | 237.06 | 11.85 |
| 4D | 30 | 223.69 | 7.46 |
| 5A | 201 | 533.14 | 2.65 |
| 5B | 232 | 417.70 | 1.80 |
| 5D | 36 | 275.62 | 7.66 |
| 6A | 169 | 406.95 | 2.41 |
| 6B | 282 | 368.72 | 1.31 |
| 6D | 185 | 893.01 | 4.83 |
| 7A | 75 | 368.03 | 4.91 |
| 7B | 220 | 440.38 | 2.00 |
| 7D | 57 | 493.35 | 8.66 |
| Whole Genome | 3197 | 9024.21 | 2.82 |

**Supplementary table S3**. Additional QTLs detected for SB after adjusting for DH and PH in the BARTAI x CIANO T79 (BC) and CASCABEL x CIANO T79 (CC) populations

| Population | Chromosome | Position | Left marker | Right marker | LOD | PVE (%) | | |
| --- | --- | --- | --- | --- | --- | --- | --- | --- |
|  |  |  |  |  |  | 2013 | 2014 | 2015 |
| BC | 4B | 349 | 2262825 | Rht-B1 | 2.94 | - | 4.86 | - |
|  | 5B | 162 | 7340609 | 976877 | 2.64 | - | - | 3.50 |
|  | 6B | 256 | 1127855 | 1291647 | 2.51 | - | - | 3.31 |
|  |  |  |  |  |  | 2014 | 2015 | 2016 |
| CC | 1A | 408 | 2257497 | 1013786 | 2.10 | 1.60 | 1.62 | - |
|  | 2B | 490 | 1167654 | 1241637 | 2.09 | 1.58 | 1.69 | 2.38 |
|  | 6D | 561 | 3064467 | K_c110911_477 | 2.25 | 2.40 | 2.39 | - |

**Supplementary table S4**. Sequence information of the GBS markers flanking the QTLs reported in Table 3 and Table S3.

| **Marker name** | **Marker sequence** |
| --- | --- |
| 976877 | TGCAGTGATGAATAAACAGAGAGGCGATTTTGGATCCCTTATGATTTGCTCGTACTTTGCTAATCTCTC |
| 1007745 | TGCAGAACTCTCCCTCTAGCGCGCATGCGCTGGTTGTGTCCACAGAGATCTTGACTGGGACCTACTACA |
| 1013786 | TGCAGCGCCATGCAGCCGAGGCCCTTGCTGTTGCGGAGGGTGGGCAGCTTGGCCGATGCCCGAGATCGG |
| 1026215 | TGCAGGTTCCAGAGGGGGCTGAAGCGGAGGCCCTTGGCGCTCATCAGGAAGCTGCGCAAGGCGGTAATG |
| 1037914 | TGCAGTGGGCGTCAAGTCCATCAGGGGTCAACCCTGGCAGTTGCAGATCAGACCGAGATCGGAAGAGCG |
| 1067537 | TGCAGTCCGTCCGTCGGAGCCATCAGATCAGAGCTCAACACGATACAGGACAGCGGGACACGAGAAAAA |
| 1072422 | TGCAGTTTTTCTTTTTGGTTCAGCACGCCGTTTAGGAGGAGCGATCAGCGCGCGCGTGAGTTATTATTT |
| 1119387 | TGCAGGCTCATCCTGGCTCCTTGATTGATGATTGTTCTTCCTTTCAGCACAAGCAATGGAGCGGGGGTC |
| 1125523 | TGCAGACCAAGATGATGAAGATGAAGGGGGTGGTGAGTGCCCAGACGGACCTCGCGGCCGAGATCGGAA |
| 1125980 | TGCAGCGCAACAAAACGCGAGTGGGTCAAGTAGTGCGCTCACTTTAGAACCATCACTGGTTGACTGTTG |
| 1126352 | TGCAGTTGCTGAACTACGCCGTTGAGAGGAGCCTTGCGGCCGACCTCGCCGTCCGTCTCTGCGGGATCA |
| 1127855 | TGCAGTTCTATGTTCAGTTCTTGTTCTTGTTATGATATGACCAGGAGAACATATTTTCATTGTCCACAA |
| 1137742 | TGCAGGGTGACAAGCAGAACCGCCCACACCTGGAAGTAGTCGTTCACCTTCACACCCGATAGTTGCGTC |
| 1167654 | TGCAGCTCCCGCCAGGGCTGTTTCCAGCATCTCGTCCGCTAGTCCATGGTGCAGCCGCCATTCCAGCAC |
| 1168776 | TGCAGAAGAAGATGAGATCCGCTCATACATCTGCATGGTCAGGCTTACTAACTATACCTGGAGCTGACA |
| 1208614 | TGCAGCTGCGAGGTGCAGCTCCGAGAGCTCCAACGGTAGCATGTGTGGTGCATGGTGAGCATGGCCGCG |
| 1218172 | TGCAGGCAGCATATATTACAAACAGCTCAGTATGAGGCATGAGCTGAGATTATATTCTCTTTCCTTAAA |
| 1241637 | TGCAGAGTGGCCATGACGCGTTTGGTTGGAATAGGTAATGTATTGGATGCTGAAGGATACAACTGTGTT |
| 1291647 | TGCAGATTTGTCGAGGGTGTGGAGTTGGACAAATGGAGGTAGCCAGGGACCATCTTAGCTAGTATAGCA |
| 1683258 | TGCAGTTGCGGAGGAAAGTGTCTGTCTGTAGAATACACTGATAAGGTTGGTGAAGTTGCTAAAACCCAT |
| 2257497 | TGCAGTGTTTCAGCTGACGGCCGTGGTCCGAACTCTTAAGTTTCTGTCTTTAGTTGTATCCCGTTCGAA |
| 2257572 | TGCAGCTAAATGCAGTAGATCAGAACCACCTAGTATTTGGACACCACTTCCACATTTCGACCTGTCCCT |
| 2262825 | TGCAGCCGTAGGCTACACAACACAGTAAAACGTTGTTGTTGTCAGTGCTCCACCAGCTGAACTTTCGCC |
| 2267710 | TGCAGACGGCATGGAGGATGCCATCGACACGTCCCAAATGTGCAAAAGGTTGTCGTTGCCACCGCTGGC |
| 3064467 | TGCAGGATGCTATGTTCGAGCCTGCCGCGGAGCCCATGGAAGAACCCAAGGCCGTATGCGTCGGTTTCC |
| 3958735 | TGCAGGTTCGCGAGCTCATATCACTCATGGCATGGCATCTTCCGAGATCGGAAGAGCGGTTCAGCAGGA |
| 4989967 | TGCAGTTTGCAGGTGGCGATCTCTCTCGAGGACCACGCGTCCATCTCCACGCAGAATTCGAAAATCCTA |
| 7340609 | TGCAGCCGCCCCGCATCGCCGAGATCGGAAGAGCGGTTCAGCAGGAATGCCGAGACCGATCTCGTATGC |
| 9724385 | TGCAGTAAAGCCACGTTTTGATGCTTGTGTGAGGGACAAGTCTTATCTCCTGTGAGACTGACCGAGGAA |
| 12002205 | TGCAGTGGAGGCAACTGTCGAGTTCCTGAACAAGGCGATGAAGCCAGTGCTTGTCGCCGAGATCGGAAG |
| 100193832 | TGCAGTTAACAGCATTATCCTCTTAATTGCCATAGTTAACAGCATGCCGAGATCGGAAGAGCGGTTCAG |
